# Resynthesis of synthetic biology techniques: combining engineered bacteria with other antitumour therapies

**DOI:** 10.3389/fmicb.2025.1545334

**Published:** 2025-07-28

**Authors:** Xueke Chang, Xiaolin Liu, Xiumei Wang, Lin Ma, Jing Liang, Yan Li

**Affiliations:** ^1^Department of Oncology, Shandong Provincial Qianfoshan Hospital, Shandong Lung Cancer Institute, The First Affiliated Hospital of Shandong First Medical University, Jinan, China; ^2^The Affiliated Huaian No.1 People’s Hospital of Nanjing Medical University, Jiangsu, China; ^3^Yuncheng Chengxin Hospital, Heze, Shandong, China

**Keywords:** engineered bacteria, synthetic biology, antitumour therapy, therapeutic effect, spatiotemporal manipulation

## Abstract

Worldwide cancer mortality rates underscore the pressing need to identify and develop novel anticancer therapies to supplement traditional cancer treatments. Naturally occurring bacteria are ideal for cancer therapy owing to their autonomous propulsion and hypoxia-targeting properties, but their poor tumour targeting ability and weak tumour penetration limit their use. Bacteria can be modified by bioengineering and nanotechnology methods to improve their physiological activity and therapeutic effect. Furthermore, engineering allows for refined spatiotemporal control, precise functional recombination, and direct genetic reprogramming. These engineered bacteria can produce synergistic anticancer effects upon coadministration with anticancer drug-containing nanomaterials or other therapeutic payloads. In this paper, the use of engineered bacteria combined with other antitumour therapies, such as radiotherapy (RT), chemotherapy, immunotherapy, light therapy and life technology, is reviewed to aid in improving antitumour therapy efficacy. In addition, we provide an overview of the current state of spatiotemporally regulated bacterial gene expression and drug release, discuss the drawbacks and difficulties of employing engineered bacteria for tumour therapy, and explore potential research avenues on the basis of current advancements.

## Graphical abstract

The combination of engineered bacteria with other antitumour therapies.
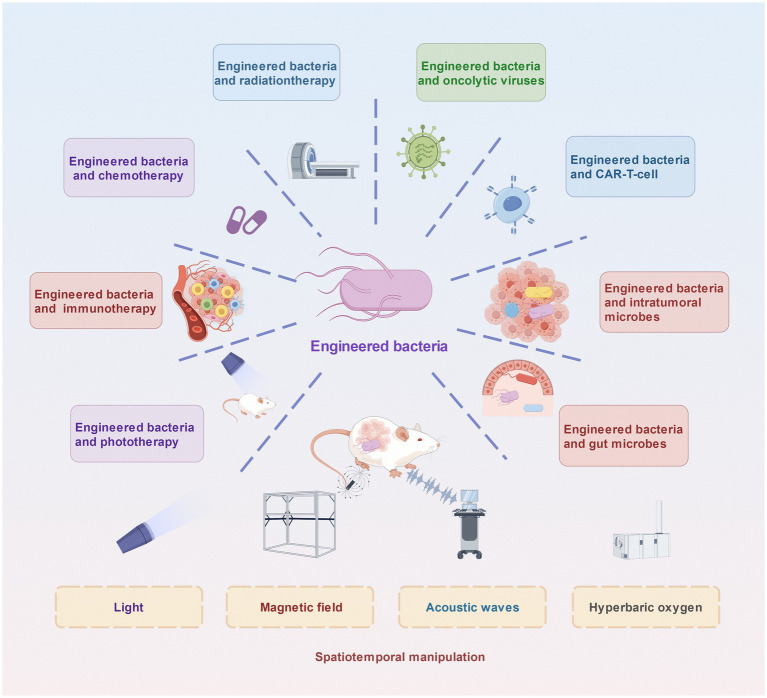

## 1 Introduction

Conventional cancer treatment methods, such as radiation therapy, chemotherapy, and surgery, are always associated with drawbacks, including a lack of specificity, development of resistance, and failure to eradicate cancer cells completely. There are potential benefits to emerging therapeutic approaches, such as immunotherapy and tumour-targeted therapy, but they also have drawbacks, such as poor clinical response rates, off-target effects, and resistance to treatment. These characteristics highlight the necessity of ongoing innovation in the cancer treatment industry (Bukowski et al., 2020; Galeaz et al., 2021; O'Donnell et al., 2019). Recently, bacteria have gained attention as potential targeted tumour therapy agents because they possess intrinsic tumour tropism, high motility, and the ability to rapidly colonise the tumour microenvironment, bypassing tumour cells and the microenvironment more rapidly than other nanostrategies that utilise peptides, aptamers, and other biopolymers. Specifically, the tumour microenvironment often presents hypoxic conditions, which predominantly favours the growth of obligate anaerobes and facultative anaerobes (Chen et al., 2023). They are more motile and chemotactic than passive diffusing nanocarriers (e.g., liposomes, exosomes) and can penetrate deeper into the hypoxic core. Bacteria amplify their numbers *in situ*, whereas nanocarriers require repeated administration. In addition, bacteria can dynamically respond to tumour conditions (e.g., hypoxia, pH) to regulate therapeutic release (Yin et al., 2025; Qiao et al., 2025). Based on these findings, researchers have focused on bacteria-based cancer treatment methods, such as intratumoral brevibacillus parabrevis enhances antitumor immunity by inhibiting natural killer (NK) cell ferroptosis in hepatocellular carcinoma (Pan et al., 2025) and *Salmonella* cancer therapy metabolically disrupts tumours at the collateral cost of T cell immunity (Copland et al., 2024), to improve the effectiveness of cancer treatment and reduce side effects, providing new modalities and directions for the development of precise tumour treatments. Unlike traditional drug carriers, bacteria’s special capacity to continuously multiply, translocate, and deliver therapeutic payloads in cancerous tissue necessitates strong and timely control of bacterial pharmacokinetics *in vivo*.

Despite demonstrating significant potential for tumour-targeted therapy, notable disparities exist between the inherent qualities of natural bacteria and an optimal tumour treatment platform (Zhou et al., 2018). As common antigens, bacteria are fundamentally harmful and can enter the human body and induce deadly side effects, including severe cytokine storms (Yang et al., 2021). Making genetic knockouts of immunogenic bacterial surface antigens, such lipopolysaccharide (LPS), is one way to get around the immunogenicity and toxicity of living bacterial therapy. However, as demonstrated by clinical trials of bacterial cancer therapy (Toso et al., 2002; Low et al., 1999; Wang et al., 2016), this approach may lead to reduced colonisation and a decrease in persistent strains. The synthetic coating of microbial surfaces with chemicals such as alginate (Low et al., 1999; Wang et al., 2016), chitosan (Low et al., 1999), polydopamine (Mitchell et al., 2021), lipids (Lu et al., 2021; Sieow et al., 2021; Brown et al., 2019), and nanoparticles (Zhao et al., 2005) is an alternate technique because surface modification has been widely employed as in concealing drug delivery vehicles (Mitchell et al., 2021). Because they prevent *in situ* regulation, these one-time, static bacterial alterations can result in unchecked growth, off-target tissue toxicity, or decreased cellular function, all of which reduce efficacy. Therefore, one of the main challenges for the practical translation of live microbial therapy in cancer is improving bacterial delivery without sacrificing safety. Furthermore, because natural bacteria have simple metabolic processes, erratic physiological behaviours, and inadequate therapeutic roles, their antitumour properties must be enhanced to satisfy treatment demands. To increase the efficacy of naturally sourced bacteria in antitumour treatment, deliberate and strategic manipulation is considered indispensable for the modification of bacterial functions (Lu et al., 2021). Owing to rapid advancements in fields such as bioengineering technology and nanotechnology, the genetic makeup of bacteria can be edited to manipulate their inherent structures, functions, characteristics, and behaviours to modulate virulence (Sieow et al., 2021; Brown et al., 2019; Zhao et al., 2005). Moreover, scientists have discovered that reediting bacterial genetic material not only can reduce bacterial virulence but can also be utilised to transform tumour-targeting bacteria into therapeutic payload carriers (Zhou et al., 2018).

Synthetic biology can enhance bacterial cancer treatment safety and efficacy through precise interactions with other cells and technologies. For example, engineered bacteria enhance radiation therapy by targeting tumour hypoxia, delivering radiosensitizers, and bridging radiation with immunotherapy via bacterial components to stimulate systemic antitumor immunity (Pan et al., 2022). Moreover, the safety and efficacy of antitumour therapies based on chemotherapy and engineered bacteria are potentiated by the configuration of the drug nanocarrier, including liposomes, micelles, and polymer nanoparticles, using synthetic biology technologies. In addition, multiple studies have shown that the inherent time structure of immune responses may be utilised to regulate the interactions between bacteria and immune cells. Throughout each phase of the biological reaction, the bacteria are capable of generating various bioactive payloads that engage with distinct subsets of immune cells, thereby facilitating a more potent reprogramming of the antitumour response. Photothermal therapy (PTT) employs photothermal agents to convert light energy into heat for tumour ablation, while photodynamic therapy (PDT) utilises photosensitizers to generate reactive oxygen species (ROS) that induce oxidative stress. Both strategies can be combined with bacterial vectors to enhance tumour targeting and therapeutic efficacy, often synergizing with immune activation for improved antitumor effects. Therefore, bacteria-based phototherapy also has great potential for future cancer treatments. Engineered bacteria in combination with RT, chemotherapy, immunity, and phototherapy may lead to new strategies for improving antitumour efficacy.

In addition to combining nonliving materials and external technologies, scientists are investigating how bacteria interact with other living cellular modules. In general, bacteria can reshape the tumour microenvironment (TME) to render it more favourable for other microbial and cellular therapies, such as the combined administration of engineered bacteria and oncolytic viruses. Bacteria can deplete antiviral cytokines in the TME and enhance the replication of oncolytic viruses; thus, they are more conducive to combined treatment with oncolytic viruses (Sun et al., 2022; Cronin et al., 2014). Bacterial administration in combination with chimeric antigen receptor (CAR)-T cells therapy can be induced by bacterial adjuvants and respond to synthetic antigens (Vincent et al., 2023). The manufactured bacterial complex can also cooperate with intratumoral bacteria and human body microorganisms, especially those in the intestines, to promote predictable immune responses or decrease the number of germs that cause cancer.

To further improve the safety and accuracy of antitumour therapy on the basis of engineered bacteria, researchers have focused on the spatiotemporal manipulation of engineered bacteria. In recent years, engineered bacteria have been combined with manipulated particles or cells, including optical (Diekmann et al., 2016; Zhang and Liu, 2008), magnetic (Carlsen et al., 2014; de Lanauze et al., 2013), and acoustic forceps (Hammarstrom et al., 2012; Gutierrez-Ramos et al., 2018), and hyperbaric oxygen (HBO) (Wu et al., 2018), providing new methods for the spatiotemporal manipulation of engineered bacteria. Engineered bacteria, when combined with multimodal therapies like radiotherapy, chemotherapy, immunotherapy, HBO and so on, significantly enhance tumour targeting and therapeutic efficacy while enabling spatiotemporal control over drug release.

## 2 Genetic engineering modification of bacteria and deep optimisation of synthetic gene pathways

The strong virulence of naturally occurring bacteria is one of the challenges for bacterial tumour therapy, and how to balance the efficacy of bacterial therapy with excessive toxicity is a dilemma. The virulence of bacteria can be weakened, their tumour targeting ability can be increased, and the efficacy of bacterial therapy can be improved by modifying their related genes. For example, the absence of msbB expression in attenuated *Salmonella typhimurium* (*S. typhimurium*) VNP20009 altered the structure of lipid A and reduced levels of bacteria-induced TNF-*α*. The secretion of a rationally designed “obligate” anaerobic *S. typhimurium* strain (YB1) can inhibit the growth of various tumours (Zhou et al., 2016; Liu et al., 2016). On the basis of the attenuated *S. typhimurium* SL7207 strain, a specialised anaerobic *S. typhimurium* YB1 was further modified. The expression of the essential gene aspartate semialdehyde dehydrogenase (ASD) is regulated by the design of the hypoxia promoter PepT and the antisense aerobic promoter SodA. ASD is a key enzyme in the synthesis of diaminohexamic acid (DAP), which is an important component of the cell walls of gram-negative bacteria and performs lysis in a short period. Under normoxic conditions, the ASD gene is not expressed, leading to DAP deficiency. Therefore, YB1 can only survive under anaerobic conditions (oxygen volume fraction <0.5%) (Wang et al., 2022; Li et al., 2017). Engineered bacteria can effectively express antitumour molecules and significantly inhibit the growth of various tumours as carriers that express nanoantibodies to block immunosuppressive signals and enhance antitumour immune responses (Gurbatri et al., 2020; Leventhal et al., 2020; Canale et al., 2021). Although the virulence of wild bacteria can be reduced by modifying their related genes and attenuated bacteria can selectively colonise tumour tissue while inhibiting tumour growth, the genetic instability of bacteria in the body may lead to ineffective or harmful phenotypes. In addition, potential antibiotic resistance or mutations that reverse attenuated bacterial phenotypes may pose a threat to patient health (Zhou et al., 2018; Kramer et al., 2018). Therefore, developing a wider and safer bacterial treatment plan is an important development direction for tumour treatment. The ongoing development of synthetic biology provides a theoretical basis and technical support for further optimisation of bacteria. On the basis of traditional bacterial modification, many important genetic pathways have been designed, including suicide switches, quorum sensing, genetic oscillators, and gating systems (Ruder et al., 2011), greatly enhancing the safety of bacteria in tumour treatment, which will lead to more clinical trials of bacterial therapy for tumours (Ruder et al., 2011).

## 3 Combination therapy with engineered bacteria and other therapies

**Figure 1:**
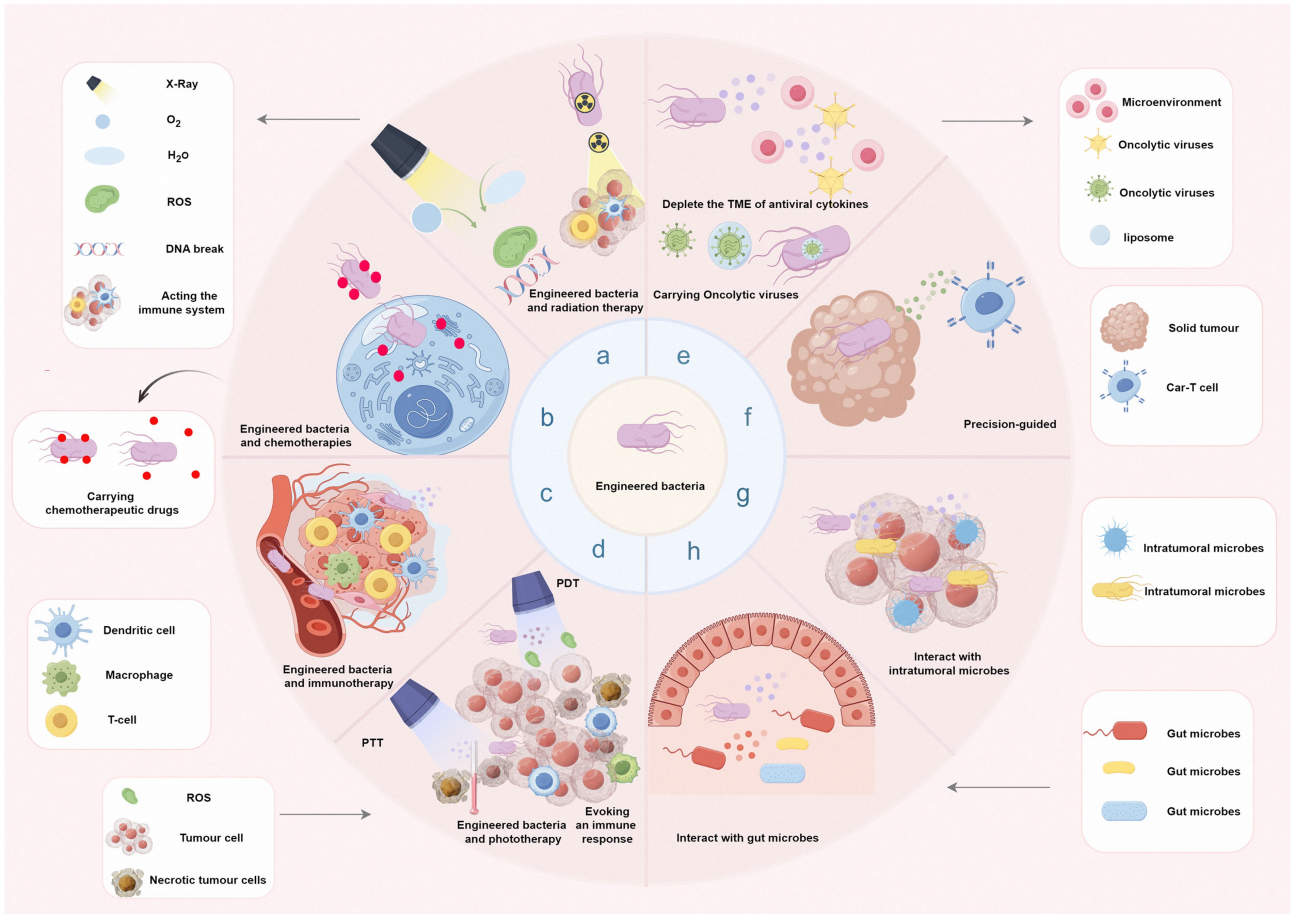
Combination therapy with engineered bacteria and other therapies. (a) Combining engineered bacteria with radiotherapy can alleviate intratumor hypoxia and increase tumour radiosensitivity to activate immunity. (b) Engineered bacteria combined with chemotherapy can be used to configure drug nanocarriers via synthetic biology techniques. (c) Throughout each phase of the biological reaction, bacteria can generate payloads to interact with specific immune cells, enabling more effective reprogramming of the antitumour response. (d) The surfaces of bacteria can be modified with appropriate photothermal and photosensitisers that are converted into thermal energy (PTT) or produce singlet oxygen (PDT) when illuminated by light without compromising their ability to target tumours or even further stimulate immune responses. (e) Combined engineered bacteria can carry or increase the replication of oncolytic viruses; thus, they are more conducive to combined treatment with oncolytic viruses. (f) Administration of engineered bacteria combined with CAR-T-cell therapy can be activated by bacterial adjuvants and respond to synthetic antigens released by bacteria. (g and h) The synthesised bacterial complex can interact with microorganisms in the human body (especially in the intestines) and intratumoral bacteria to promote predictable immune responses or decrease the number of germs that cause cancer.

Bacterial monotherapy is sometimes insufficient to eliminate primary or metastatic cancer cells. Traditional anticancer drugs are spread throughout the body via the circulatory system, and their indiscriminate attacks cause many normal cells to die. The complex vascular systems of tumours hinder the profound delivery of traditional anticancer drugs, making it difficult to achieve sound tumour-killing effects. Combining the administration of engineered bacteria with traditional therapies can effectively compensate for their respective shortcomings. In addition, bacteria-based phototherapy also has excellent potential for future cancer treatment (Figure 1).

### 3.1 Engineered bacteria and radiation therapy

#### 3.1.1 Radiation therapy of engineered bacteria

Live attenuated bacteria have been successfully used in numerous trials to transport radionuclides to tumours and metastatic sites in a targeted manner, increasing therapeutic effectiveness and minimising harm to healthy tissues. For example, a distinct radioactive strain of *Listeria monocytogenes* was produced by coupling the radioisotopes ^188^Rhenium (Quispe-Tintaya et al., 2013) and ^32^Phosphorus (Chandra et al., 2017) to attenuated live Listeria. This strategy significantly reduced the growth of Panc-02 tumours. This technique produces antitumour effects without causing damage to healthy tissues.

Optimising the hypoxic environment within tumours through the use of modified bacteria has emerged as a novel approach to increase the efficacy of radiation therapy. For example, in one study, intratumoral hypoxia was reduced, and tumour radiosensitivity was increased by the use of a genetically modified form of the catalase-secreting, tumour-targeting probiotic EcN bacteria (Huang et al., 2021). Pan P et al. developed an integrated nanosystem (Bac@BNP) that consists of engineered bacteria (Bac) and BNPs for radiosensitisation. Because BNPs can cause DNA damage in specific tumour cells and increase the intracellular generation of reactive ROS, they can improve the effectiveness of RT when exposed to X-ray radiation (Pan et al., 2022). Bettegowda C et al. treated transplanted malignancies in mice using *Clostridium novyi*-NT spores. In a number of the examined mouse models, the bacteria were found to Overcome the hypoxic barrier to radiation therapy and significantly increase the effectiveness of radiation (Bettegowda et al., 2003).

#### 3.1.2 Radiation immunotherapy of engineered bacteria

Many factors affect tumour growth and the response to RT, among which immune regulation is essential. Therefore, some engineered bacteria, their components, products, and inactivated bacterial carriers serve as bridges, cleverly linking RT and immunotherapy and providing inspiring ideas for the multiefficacious immune activation function of bacterial carrier-mediated radiation immunotherapy. Chen MH et al. successfully synthesised a unique and complex compound, termed Au-OMV, which consists of two specific types of nanoparticles: gold nanoparticles and outer-membrane vesicles (OMVs) derived from *Escherichia coli*. Au-OMV showed strong immunomodulatory and radiosensitising effects when paired with radiation therapy, successfully inhibiting the growth of tumours in mice (Chen et al., 2021). Pei P et al. created an inactivated bacterial vector with ^125^I/^131^I labelling (^125^I-VNP/^131^I-VNP), retaining radioiodine at the tumour site and delivering it to tumour cells and TAMs, thereby achieving successful and harmless internal radioisotope therapy (IRT). Local IRT by ^131^I-VNPs further stimulated systemic antitumour immunity by bolstering innate immunity and promoting DC maturation for T-cell-dominated immunity. Combining this therapy with systemic checkpoint blockade therapy (αPD-L1) can upregulate PD-L1 expression in distal tumours, inhibit local colon cancer and protect against tumour rechallenge (Pei et al., 2022). Radiation immunotherapy might benefit greatly from the engineering of bacteria and some processed biomaterials produced from microorganisms, which can increase radiation sensitivity by altering the TME and improving radiation deposition at tumour locations. Although there are many opportunities to modify bacteria for use in conjunction with radiation therapy, further research is needed to determine their biological safety and mode of action.

### 3.2 Engineered bacteria and chemotherapy

Chemotherapy disrupts DNA integrity and repair/synthesis enzymes but has limited efficacy due to resistance/toxicity (Ting et al., 2022; MacDiarmid et al., 2007). Nanocarriers can be used to deliver drugs, but the targeting ratio is low (Wilson and Hay, 2011). Conventional delivery systems, such as liposomes, micelles, and polymeric nanoparticles, are often hindered by physical and chemical barriers that prevent them from reaching hypoxic regions. In contrast, bacteria-based delivery systems can navigate through these barriers and selectively colonise hypoxic tumour tissue (Loeffler et al., 2008; Ganai et al., 2011; Swofford et al., 2015; Zoaby et al., 2017), thereby improving the therapeutic effect of combined chemotherapy (Yang et al., 2023).

#### 3.2.1 Engineered bacteria delivering liposomes

Liposomes, which are used for antitumour drug loading, can serve as microcarriers for engineered bacteria. For example, O. Felfoul et al. reported that the magnetoaerotactic migration behaviour of the magnetotactic bacteria *Magnetococcus marinus* strain MC-14 could be used to deliver drug-loaded nanoliposomes into the hypoxic regions of the tumour (Felfoul et al., 2016). Similarly, *S. typhimurium* can also deliver paclitaxel-loaded liposomes more effectively because of its rapid movement and tumour tropism, which improve its antitumour effects (Zoaby et al., 2017; Nguyen et al., 2016).

The challenge is the loading of drug-carrying cargos. The motion patterns of bacteria, their diffusion out of blood arteries, and their penetration into solid tumours are all impacted when cargoes of typical sizes of several hundred nanometers are loaded. For efficient passage, flagellated bacteria with a diameter of roughly 1–2 μm are best. Due to the small openings in the interstitial spaces of solid tumours and leaky tumour arteries, the navigation efficiency will be impacted by the notable increase in bacterial size upon cargo loading (Taherkhani et al., 2014).

#### 3.2.2 Engineered bacteria delivering micelles

In 2018, scientists conducted an study into a new species of bacterial microbot that paired micelles with bacteria, resulting in an exceptionally effective antitumour effect. This unique approach was found to enhance antitumour efficacy by simultaneously releasing doxorubicin (DOX) into the nucleus and *α*-tocopheryl succinate into the mitochondria (Xie et al., 2018). In the face of a slightly acidic tumour environment, precursor micellar copolymers were released from the bacteria and self-assembled into micelles, and the released heterogeneous micelles presented cellular uptake efficiencies, glutathione-sensitive drug release, and cytotoxicity similar to those of micelles prepared by solvent evaporation. Upon intravenous administration to tumour-bearing mice, the engineered bacteria selectively accumulated in the tumour and prolonged drug retention in the tumour region. The antitumor effect was significant and less toxic because of the continuous process of self-propulsion of the bacteria themselves and preferential accumulation of bacteria in the tumour, *in situ* formation of heterogeneous micelles, and intracellular drug release. Thus, a novel bacterial microrobot has been shown to load, deliver and release active drugs for synergistic cancer therapy (Xie et al., 2018).

#### 3.2.3 Engineered bacteria delivering polymeric nanoparticles

Research has demonstrated that DOX-linked glycerol nanoparticles can successfully lower the toxicity of DOX (Zhou et al., 2020). However, a drawback of most nanoparticles is their propensity to opsonise, which allows them to pass quickly via the liver and spleen’s reticuloendothelial system (Lesniak et al., 2013). It is critically important to modify nanoparticles to minimise opsonisation, extend the circulation time and enhance tumour targeting. Conventional chemical modifications have limitations in effectively treating cancer. However, recent developments in genetic engineering technology have created new opportunities for the combined use of chemotherapy and nanoparticles in the treatment of cancer. Suh et al. effectively developed an advanced nanoscale bacteria-enabled autonomous drug delivery system (NanoBEADS). With no outside stimulation, this cutting-edge method can transport more than 100 times as many nanoparticles to solid tumours (Suh et al., 2019). In subsequent research, anaerobic *Bifidobacterium infantis* was utilised to deliver adriamycin-loaded bovine serum albumin nanoparticles (Xiao et al., 2022) and polydopamine (PDA)-coated paclitaxel nanoparticles (Shi et al., 2023) specifically into tumour tissues. These autonomous biohybrid systems unlock new cancer treatment paradigms by enhancing chemotherapeutic drug efficacy and minimising side effects. Another important development was a 2022 study that combined magnetic nanoparticles and nanoliposomes loaded with chemotherapeutic compounds (DOX) and photothermal agents (indocyanine green) onto *E. coli* with an efficiency of approximately 90%. This method produced a biological hybrid of bacteria that was magnetically manipulated (Gwisai et al., 2022). According to a new study, doxorubicin nanoparticles along with engineered bacteria can successfully inhibit angiogenesis and metastasis in murine melanoma models (Yang et al., 2023). Engineered bacteria delivering inorganic nanomaterials is also an ingenious idea. Li F et al. modified DH5α *E. coli* and further surface armored it with iron-doped ZIF-8 nanoparticles. This bioengineered *E. coli* was able to respond in a hypoxic tumour microenvironment by producing and secreting lactate oxidase to reduce lactate concentration and trigger immune activation. The peroxidase-like function of the nanoparticles prolongs the end product of lactate metabolism, allowing the conversion of hydrogen peroxide into highly cytotoxic hydroxyl radicals. Furthermore, in combination with the conversion of tirapazamine uploaded on the nanoparticles into toxic benzotriazinyl, it ultimately leads to severe iron death of tumour cells. Intravenous administration of this biocomplex significantly inhibited tumour growth and metastasis (Li et al., 2024).

#### 3.2.4 Bacterial derivatives carrying drug

Apart from whole bacteria, numerous bacterial derivatives, including minicells, OMVs, and bacterial protoplast-derived nanovesicles, have demonstrated drug-carrying capabilities and are now the subject of extensive investigation (MacDiarmid et al., 2007; Flemming, 2007; Jivrajani and Nivsarkar, 2016; Solomon et al., 2015). Kim et al. created EGFR-expressing *E. coli* protoplast nanovesicles, which efficiently delivered drugs to tumour cells, suppressing growth without harmful effects (Kim et al., 2017). A recent study demonstrated that doxorubicin-loaded bacterial OMVs/DOX can be selectively recognized by neutrophils, thereby facilitating glioma-targeted delivery of drugs, resulting in significantly increased tumour accumulation (by 18-fold) compared with conventional passive targeting mechanisms (Mi et al., 2023).

### 3.3 Engineered bacteria and immunotherapy

#### 3.3.1 Innate immunity programming

**Figure 2:**
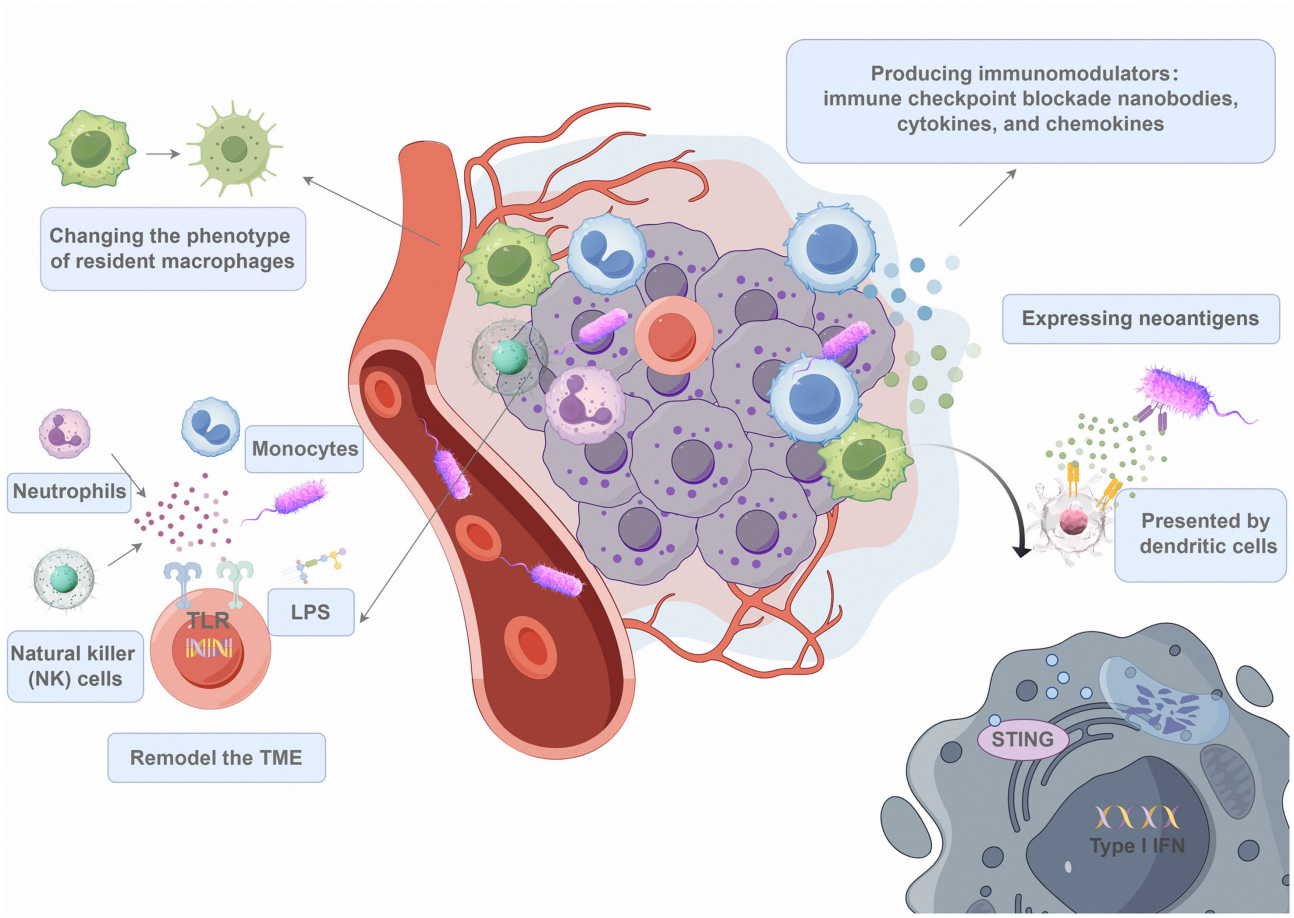
Engineered bacteria and immunotherapy. Immunogenic single-agent bacteria can change the TME by binding to TLR-4 and TLR-5, which are activated by flagella and lipopolysaccharide, respectively. As a result, the native macrophage phenotype changes, and innate immune cells such as neutrophils, monocytes, and NK cells infiltrate the tumour. Bacteria are phagocytosed as immune cells move into the malignant region, providing a chance to transfer immune cell-specific cargo intracellularly. As a result, STING agonists can be delivered to intratumoural APCs by *E. coli*, triggering IFN-I reactions. By generating immunomodulators (such as cytokines and chemokines) to attract tumour-infiltrating lymphocytes to the tumour area, bacteria can engage with the host’s adaptive immune system.

Studies have recently probed bacterial delivery systems in combination with immunotherapy, attracting significant interest from researchers and clinicians alike. Multiple studies have shown that the inherent time structure of immune responses may be utilised to regulate the interactions between bacteria and immune cells. By producing payloads to engage certain immune cells at each stage of the reaction, bacteria can more successfully rewire the antitumour response (Figure 2). As single agents, bacteria can engage Toll-like receptor 4 (TLR-4) and Toll-like receptor 5 (TLR-5), which are activated by flagella and lipopolysaccharide, respectively, to alter the TME. Innate immune cells such as neutrophils, monocytes, and NK cells may infiltrate the tumour as a result, and the phenotype of resident macrophages in the tumour microenvironment may be altered (Zheng et al., 2017). However, Flagellin B (FlaB) failed to attract and activate enough NK, CD4, CD8, and NKT cells in tumour tissue; therefore, Zhang Y et al. designed a synergistic cancer immunotherapy system that utilises programmed *S. typhimurium* to secrete heterologous FlaB conjugated to interleukin-15 proteins to compensate for this deficiency (Zhang et al., 2023). Bacteria are phagocytosed by immune cells as they enter the malignant area, which opens the door for the intracellular delivery of immune cell-specific cargo. For example, stimulator of interferon genes (STING) agonists can be delivered to intratumoral antigen-presenting cells (APCs) via *E. coli*, which triggers IFN-I responses. Phase I clinical trials are currently testing this strategy in patients with lymphoma or advanced solid tumours (NCT04167137) (Leventhal et al., 2020). Considering the tumour penetration effect and high biocompatibility of EcN as a delivery vector, we subsequently developed a bacterial system for stinger amplification by combining a stinger agonist with a photothermal agent (polydopamine nanoparticles), which, following irradiation, produced yiIFN-i-mediated reactions in the near infrared (NIR) region. Both its spatiotemporal position control and anoxic tumour tissue penetration were preserved, and it demonstrated good photothermal characteristics (Chen et al., 2023). Xie X et al. rescued tumour immune escape and promoted phagocytosis of tumour cells by macrophages by interfering with the interaction between CD47 on the surface of tumour cells and SIRPα on macrophages. This study enhances the regulatory role of therapeutic bacteria in tumours, improves the safety and efficacy of therapies, and also promotes the development of tumour immune checkpoint therapy in a more intelligent and personalized direction (Xie et al., 2025).

#### 3.3.2 Adaptive immunity programming

While engulfing cells, APCs have the ability to present new antigens and activate the body’s adaptive immune response (Kalaora et al., 2021; Shahabi et al., 2008; Selvanesan et al., 2022). For example, melanoma cells and invading APCs can elicit adaptive immunity by presenting antigens from local intracellular bacteria (Kalaora et al., 2021). *Listeria* (Selvanesan et al., 2022; Scanlon et al., 2023) and *S. typhimurium* (Hyun et al., 2021) have successfully become new neoantigen peptide delivery vectors. In a recent article published in Science, *Staphylococcus epidermidis* strains modified to express model antigens and tumour-derived neoantigens were shown by Chen YE et al. to evoke tumour-specific CD4 + and CD8 + T lymphocytes following colonisation (Chen et al., 2023). Similarly, Sajjath SM et al. reported that genetically modifying the skin commensal *S. epidermidis* to express a foreign antigen carried by melanoma cells can generate a powerful effector T-cell response in mice without inducing inflammation at the site of colonisation (Sajjath et al., 2023). Engineered bacteria regulate metabolism to become a better idea for anti-tumour therapy (Qiao et al., 2025; Wang et al., 2024; Wang et al., 2025). Tryptophan is an amino acid that enhances the function of CD8 + T cells. Wang et al. designed an engineered bacterium that increased the bacterial secretion of tryptophan, thereby increasing the concentration of tryptophan in the TME and ultimately improving the function of CD8 + T cells (Wang et al., 2024). Although immunotherapy is promising, its efficacy is limited by the immunosuppressive effects of the TME, in which adenosine metabolites play a key role. Wang J et al. utilised genetic engineering technology to modify the probiotic EcN to express adenosine deaminase specifically in the tumour hypoxic environment. Adenosine deaminase can effectively convert adenosine to inosine, thereby reversing the immunosuppressive effects of adenosine on CD8 + T cells and macrophages. The catalytic production of inosine by adenosine deaminase can maintain the function of effector CD8 + T cells (Wang et al., 2025).

Bacteria can also interact with the host’s adaptive immune system by producing immunomodulators (such as cytokines and chemokines) to attract tumour-infiltrating lymphocytes to the tumour space (Canale et al., 2021; Gurbatri et al., 2022; Din et al., 2016). According to a phase I clinical trial, in 22 patients with metastatic gastrointestinal cancer, oral delivery of *Salmonella* expressing human IL-2 dramatically increased the number of circulating NK and NKT cells (Gniadek et al., 2020). Tumas S. et al. subsequently carried out stepwise optimisation of biologically active IL-2 for delivery via *E. coli* Nissle 1917. They showed that the optimised strain could modify the tumour microenvironment by doubling intratumoral levels of IL-2 and reducing tumour growth rates (Tumas et al., 2023). Wu Y et al. designed an engineered bacterium capable of releasing interleukin-15 and interleukin-15 receptor *α* in response to temperature changes after microwave ablation treatment, which effectively inhibits tumour growth and prolongs animal survival even when microwave ablation is incomplete (Wu et al., 2024). Chang Z et al. activated IL-10 receptor expression in immune cells by engineered *Salmonella*, prompting tumour-associated macrophages to produce IL-10, thereby avoiding phagocytosis by tumour-associated neutrophils and simultaneously expanding and stimulating CD8 + T cells within the tumour. This mechanism effectively eliminated the tumour, prevented recurrence and inhibited metastasis. Moreover, the high expression of IL-10 receptors may be a common feature of multiple human tumour types, providing a new framework for bacterial immunotherapy in solid tumours (Chang et al., 2025). Recently, Savage TM et al. used engineered bacteria that produce tumour-localized CXCL16 and the synchronised lysis circuit (SLC) to attract CD8 + T cells and stimulate antitumour immunity. CXCL16 combines with the chemokine CCL20 to engage innate and adaptive immune cells that participate in tumour immune initiation and response stages, enhance the overall antitumour immune response, and enhance therapeutic efficacy (Savage et al., 2023). Another crucial immune-regulating molecule is the cytokine interferon-*γ* (IFN-γ). In preclinical models, Chen Y et al. created an ultrasound-responsive bacterium to regulate IFN-γ release at the tumour site, promoting cancer cell apoptosis, macrophage polarisation from the M2 phenotype to the M1 phenotype, and CD4 + and CD8 + T-cell activation, all of which enhance the antitumour immune response (Chen et al., 2022). Additionally, systemic tumour-antigen-specific immune responses are stimulated by a nanobody antagonist of CD47 (CD47nb)-expressing bacteria, which inhibits the growth of untreated tumours and provides evidence of the abscopal effect of engineered bacterial immunotherapy (Chowdhury et al., 2019). The release of cytotoxic T lymphocyte-associated protein 4 and programmed cell death ligand 1 antibodies from tumours after steady lysis by a probiotic system can result in an abscopal effect, a relative increase in activated T cells, and corresponding increases in systemic T-cell memory populations in mice given probiotic-delivered checkpoint inhibitors (Gurbatri et al., 2020). In addition to directly generating immunomodulatory cargo, engineered bacteria can also transform waste products from tumour metabolism, such as ammonia, into metabolites, such as L-arginine. By doing so, they can modify the TME and increase the frequency of TILs (Canale et al., 2021).

A progress has been reported by a research group, which indicates that the combined activation of innate and adaptive immune responses through bacteriotherapy can effectively prevent the recurrence of glioblastoma. This local bacterial therapy, designed to stimulate the immune system, holds promising potential for application in patients with malignant tumours who are at a significant risk of relapse (Zhang et al., 2024). In addition to whole cells, bacterial derivatives, such as bacterial OMVs and multifunctional bacterial membrane-coated nanoparticles (BNPs), have also been developed and applied in immune-related antitumour treatments (Chen et al., 2020; Patel et al., 2019).

### 3.4 Engineered bacteria and phototherapy

Recently, advancements in phototherapeutic modalities, including PTT and PDT, have garnered significant attention. These techniques utilise separate photothermal and photosensitising agents that are converted to thermal energy (PTT) or produce singlet oxygen (PDT) upon light irradiation, thereby inducing hyperthermia or oxidative stress in tumour cells, which subsequently destroys the tumour cells (Xie et al., 2020; Li et al., 2020). Additionally, PDT and PTT are noninvasive and have shown promise when used as monotherapies or combined with other treatments. Bacterial surfaces can be modified with suitable photothermal/photosensitive agents without impairing their ability to target tumours (Tong et al., 2024; Zheng et al., 2025).

#### 3.4.1 PTT

PTT efficacy relies on potent photothermal agents (PTAs) with high photothermal conversion efficiency. However, poor PTA tumour accumulation hampers its clinical efficacy. Anaerobic bacteria deliver nanomaterials to hypoxic tumour areas for imaging and PTT, increasing the PTA distribution into tumour necrotic and hypoxic regions (Luo et al., 2016). Chen et al. used *Salmonella* VNP20009 to deliver PDA into hypoxic tumour areas for immunotherapy and PTT, evoking an immune response by triggering tumour cell ablation and releasing antigens (Chen et al., 2019). However, traditional methods in nanotechnology, which depend on genetic manipulation, are often complex. Furthermore, these surfaces are adorned with numerous palpable nanoparticles or a thick coating of polydopamine on the bacterial membrane, which could disrupt the natural division and viability of the cells. The development of a simpler, physically nonintrusive approach to nanofunctionalise nonpathogenic natural bacteria is highly important. Consequently, nanoengineered Bifidobacteria were created by encapsulating organic dye molecules within a straightforward culture and washing process, resulting in an effective photothermal conversion and the regression of tumours in mice, which is courtesy of an immune response (Reghu and Miyako, 2022). Although these systems are promising for drug delivery, the nanomaterials on the bacterial surface are unavoidably exposed to body fluids, leading to potential nonspecific interactions with proteins and premature payload release. To address this, a “Trojan” strategy was developed, which involves the fusion of photosensitive nanopreparations with the cell membrane via ATP-binding cassette transport channels (ATP-binding cassette transporters, ABCs) into the bacterial cell, allowing for deep tumour targeting and penetration by the bacteria (Chu et al., 2022).

However, photosensitive bacteria face challenges with a single, easily decaying photothermal effect and a low photosensitiser load, leading to a high bacterial dose or radiation intensity. Therefore, monochromatic irradiation-mediated ternary combinations of photosensitisers (all components of the formed ternary combination share sufficient excitation at 808 nm) have been developed for photoacoustic imaging-guided synergistic photothermal therapy to produce homogeneous imaging signals and sufficient tumour hyperthermia at a safe dose (Guo et al., 2023). In addition to the development of innovative photosensitisers, ongoing research is needed to identify safer and more efficient bacterial vectors. Consequently, EcN, a probiotic with quick metabolism and simple removal from healthy tissues and known for its rapid metabolism and easy elimination from normal tissues, has been developed. It exhibits excellent photothermal properties and can activate antitumour immunity (Yao et al., 2022). Shortly thereafter, purple photosynthetic bacteria (PPSB) were employed for tumour targeting, offering unique advantages such as near-infrared (NIR) fluorescence, strong photothermal conversion ability, high biocompatibility, and immune regulation. When stimulated by NIR irradiation, these functionalised PPSBs exhibit specific tumour-targeting NIR fluorescence and significant anticancer effects (Reghu et al., 2023).

OMVs from gram-negative bacteria are used as adjuvants or antigens for antibacterial treatments. Recently, they have also been tested in antitumour treatments. An i.v. injection of OMVs activates the immune system by increasing antitumour cytokine levels and promoting red blood cell extravasation into tumours, causing tumour darkening and increasing NIR absorbance, enabling photothermal ablation (Zhuang et al., 2021). Li et al. developed nanopathogens (NPNs) that are encapsulated within bacterial OMVs, which are internalised by neutrophils. Neutrophils transport the NPNs to residual microtumours, allowing the NPNs to home in and achieve complete eradication of the tumours after phototherapy (Li et al., 2020).

#### 3.4.2 PDT

Because PDT is less invasive, has fewer side effects, and lowers the chance of drug resistance, it has been widely accepted in the treatment of cancer. It mainly uses ROS produced when photosensitizers (PSs) are photoactivated to cause necrosis and apoptosis in cells, which promotes tissue destruction. However, bacterial PS delivery can address the constraints of PDT efficacy caused by the hypoxic TME, in which the high oxygen demand of PSs worsens hypoxia in the tumour (Wu et al., 2019). For example, Shi L et al. developed a strategy in which engineered oncolytic bacteria (*Clostridium butyricum*) metabolise d-alanine (d-Ala) to form d-Ala-TPAPy (d-alanine photosensitiser), which is incorporated into the bacterial peptidoglycan structure, resulting in engineered *C. butyricum*. Once injected into the melanoma site, the bacteria thrive only in the anaerobic tumour region, triggering an immune response and inhibiting hypoxia, causing increased oxygen levels in the tumour periphery and bacterial death. The photosensitiser (PS) initiates a photodynamic reaction in the oxygen-rich region, leading to further destruction of the melanoma (Shi et al., 2022). Recently, Chen B et al. designed a living system formed by genetically engineered *E. coli* MG1655 cells expressing tumour necrosis factor-related apoptosis-inducing ligand (TRAIL) associated with black phosphorus (BP) nanoparticles (NPs) to form the Ec-T cells associated with BP NPs on their surface. This system can remove photoelectrons from BP NPs to reduce nitrate to nitric oxide at tumour sites and improve therapeutic efficacy and macrophage polarisation. Moreover, the generation of ROS can induce the death of immunogenic cells, thus promoting increased antitumour efficacy (Chen et al., 2023). Lin W et al. developed a photosensitiser–bacteria hybrid, iridium (III) photosensitiser-bacteria hybrid (Ir-HEcN), which can cause immunogenic cell death and pyroptosis in tumour cells under irradiation; Ir-HEcN is the first metal complex-adorn bacteria used for improved photodynamic immunotherapy (Lin et al., 2023).

#### 3.4.3 Optogenetics

Recently, light-regulated modules have garnered much attention because of their capacity to influence cellular or molecular behaviour. Among these methods, optogenetics uses genetics and optics to quickly activate or deactivate photosensitive proteins. This technique has much potential for controlling gene transcription, neuronal activity, and cellular functions in living organisms. Zhu X et al. paired EcN with lanthanide upconversion nanoparticles after modifying it to detect and release flagellin B in response to blue light. The fact that bacteria release flaB when exposed to illumination at 808 nm, which binds to Toll-like receptor 5 expressed on macrophages, resulting in an immunological response and tumour regression, demonstrates the potential of optogenetic techniques and bacteria for cancer immunotherapy (Zhu et al., 2023).

#### 3.4.4 Combining multiple antitumour approaches

A team described an upconversion double photosensitiser--expressing bacteria used for NIR monochromatically excitable synergistic PTT and PDT, showing better treatment prospects than a single phototherapy strategy (Chen et al., 2024). Some studies have reported that engineered bacteria (EcN-cypate)-mediated PTT and HBO (and anti-PD-1)-enhanced systemic immune responses promote tumour eradication (Xu et al., 2024). These findings suggest that combining multiple antitumour approaches may have unexpected effects.

### 3.5 Combining engineered bacteria with life technology

In addition to combining nonliving materials and external technologies, scientists are investigating how bacteria interact with other living cellular modules. In contrast to external technologies and nonliving materials, which require external actuation, engineer interactions between bacteria and other living cellular modalities can perform complex biological tasks autonomously; and they can interact dynamically with the TME, making them more adaptable than inanimate tools that operate through fixed parameters.

#### 3.5.1 Engineered bacteria and oncolytic viruses

Currently, oncolytic viruses (OVs) have been extensively researched for their ability to treat cancer because of their capacity to trigger a systemic immune response, particularly during the replicative phase of the virus. However, the limited immune activation and lack of OV enrichment within tumours have hindered the extensive therapeutic use of OVs. To enhance immunotherapy for cancer, Sun M et al. suggested the idea of bacteria-assisted OV targeting to tumours via liposome-cloaked oncolytic adenoviruses (OAs) conjugated to tumour-homing *E. coli* BL21 (referred to as *E. coli*-lipo-OAs) (Sun et al., 2022). Furthermore, bacteria can alter the TME, creating a microenvironment that is deprived of bioactive antiviral cytokines. This “preconditions” the tumour to improve the ability of the OV to destroy it later (Cronin et al., 2014).

#### 3.5.2 Engineered bacteria and CAR-T cells

CAR-T-cell therapy is a promising type of tumour antigen-targeting therapy for treating cancer, but finding suitable antigen targets that are specific and uniformly expressed on heterogeneous solid tumours is a core challenge. Vincent RL et al. developed a probiotic-guided CAR-T-cell platform that specifically colonises tumours with engineered probiotics and releases synthetic antigen-labelled tumour tissue, providing precise guidance for CAR-T cells to recognise and kill tumours. This strategy overcomes the bottleneck of CAR-T-cell therapy for solid tumour treatment and has shown good therapeutic effects in mouse models and clinical translational potential (Vincent et al., 2023).

#### 3.5.3 Engineered bacteria and microbial communities

In addition to cell therapy, the human body (especially the intestine) and tumours also contain many microbial communities, which can be used to eliminate metabolic waste, improve the internal environment of the body, and achieve the goal of treating diseases. Recent advances in engineered probiotics demonstrate their potential in ameliorating ulcerative colitis through modulation of gut microbiota and redox homeostasis (Guo et al., 2024). Tang He et al. constructed an engineered probiotic, Ep-AH, to simultaneously express HlpA and azurin on the basis of a targeted killing strategy. Ep-AH not only has excellent antitumour effects on azoxymethane/dextran sodium sulfate salt-treated mice but also improves intestinal microbiota dysbiosis and abnormal metabolic function. Consequently, the modified probiotic Ep-AH may be a viable option for the microbial treatment of colorectal cancer (CRC) (Tang et al., 2023).

Tumours carry bacteria, which increases the likelihood that the microbiome is actively involved in tumour occurrence, development, and treatment. Intratumoral bacteria, as attractive molecular targets for tumour treatment, hold great promise in improving cancer therapy. Therefore, efforts have been made to observe whether antitumour bacteria can be modified to treat cancer. Menglin Wang et al. used liposome antibiotics to kill tumour-associated bacteria to generate neoantigens, thereby inducing an antitumour immune response (Wang et al., 2024). However, the direct use of antibiotics to regulate the bacterial community and other *in vivo* antimicrobial strategies to achieve cancer treatment gains are often uncertain, mainly due to a lack of antibiotic selectivity, the inability to distinguish between beneficial and unfavourable bacteria, and the potential for adverse reactions. Thus, commensal bacterial-derived extracellular vesicles with responsive nanocloaks have been designed to enhance the strong immune response to tumour growth and metastasis progression by developing an activatable biointerface (Zhang et al., 2024).

Even while individual bacteria can be precisely controlled through engineering, their complicated applications are severely limited by their unicellular reproduction and excessive genetic features. In general, homogeneous bacteria have a common nutritional source, metabolic processes, and metabolites. As a result, they are more brittle and unstable in complicated settings. Like the numerous cells that form living organisms, bacteria are also able to gather to form communities to improve their survival. Owing to the interaction between communities and the environment, colonies of bacteria are highly adaptable, more organised, and better able to respond to changes in the environment than individual bacteria. Strong synthetic communities with antagonistic or cooperative symbioses can be created through the use of synthetic biology techniques to enhance therapeutic results (Liao et al., 2019; Karkaria et al., 2021; Kong et al., 2018; Gao et al., 2023). However, the stability of microbial communities remains an unresolved issue for biotechnology applications. Engineered combinations exhibit significantly different behaviours on the basis of starting conditions, requiring better control and accurate measurement methods. In addition, the increase in complexity of microbial communities may disrupt their stability, reducing their inherent stability and ability to withstand disturbances. Recently, Wang L et al. proposed a method and strategy for artificial spatial isolation that flexibly and accurately constructs stable microbial communities across species. The controllable assembly of various synthetic microbial communities was achieved, resulting in the biosynthesis of 34 enzyme systems, the biodegradation of pollutants, the division of labour and communication between microbial communities across species, and the construction of photosynthetic microbial communities (Wang et al., 2022). An engineered microbial consortia was used to design and produce a photoautotrophic “living material” for Promoting Skin Wound Healing. It was demonstrated that these modified microorganisms in the “living material” operated steadily for a longer period of time than merely individual germs (Li et al., 2023). Zhou T. et al. formed a bacterial cooperative to target colorectal cancer. The bacterial consortium members improved intestinal dysbiosis and metabolic dysfunction, correctly recognized tumour microenvironmental traits *in vitro*, and prevented the formation of subcutaneous tumours in mice (Zhou et al., 2024). Therefore, the coordination and integration of multiple bacterial groups can provide more complex and delicate functions than individual bacterial modifications. This represents a unique and promising development in antitumour platforms. However, retaining stability between different strains as well as the proportion of reproducible colonies remains a significant challenge, limiting the full therapeutic potential of engineered colonies. The evolution of manufactured colonies in the future will likely follow these goals.

## 4 The spatiotemporal manipulation of engineered bacteria

**Figure 3:**
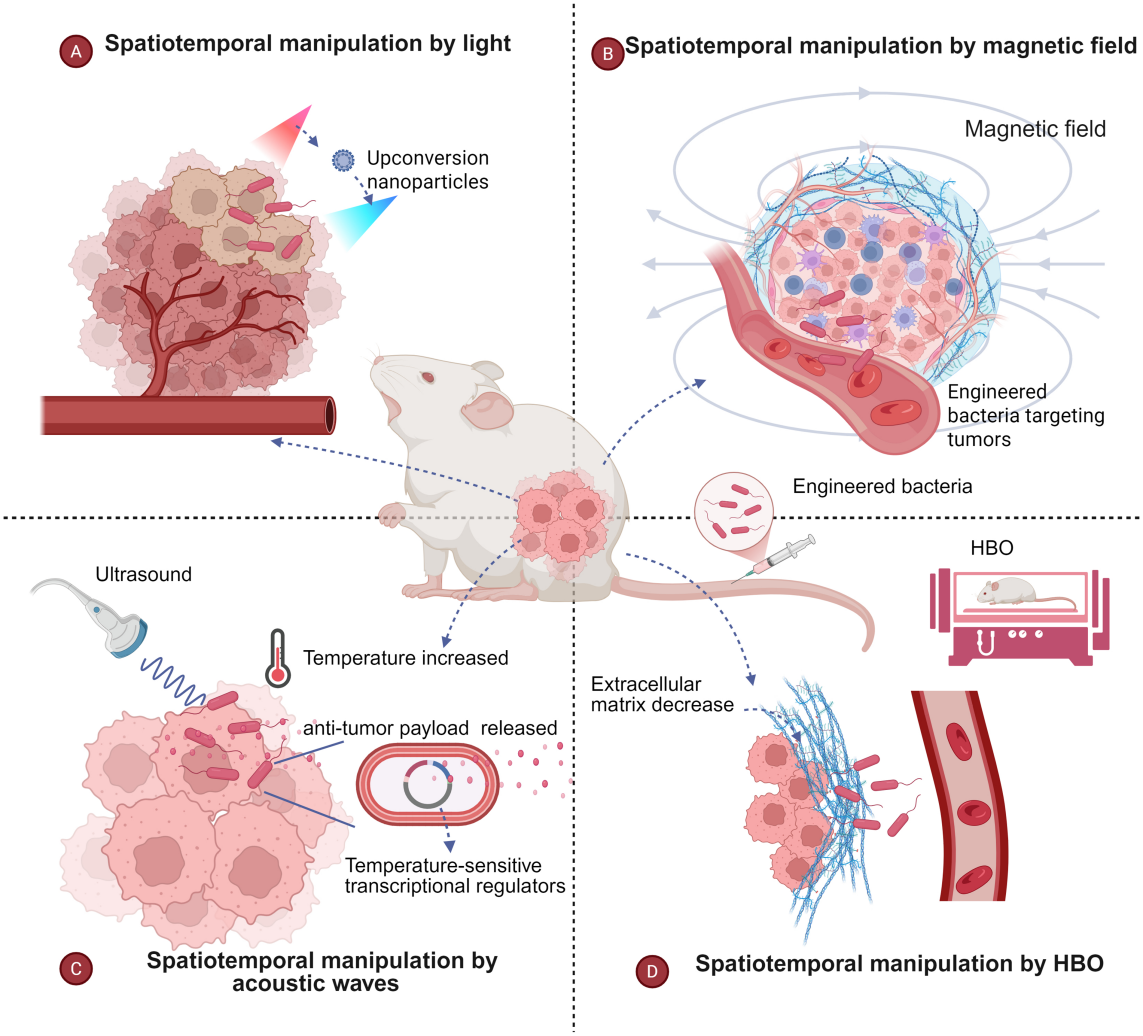
The spatiotemporal manipulation of engineered bacteria. **(A)** Spatiotemporal manipulation by light; **(B)** spatiotemporal manipulation by a magnetic field; **(C)** spatiotemporal manipulation by an acoustic wave. **(D)** Spatiotemporal manipulation by HBO. Created in BioRender. Chang (2025): https://BioRender.com/1ao7wx5.

The use of engineered bacteria in conjunction with other treatments has advanced significantly, significantly increasing the effectiveness of antitumour treatments. To further increase the precision and safety of bacterially engineered antitumour therapy, researchers have focused on the spatiotemporal manipulation of engineered bacteria (Figure 3). Numerous technologies have been created thus far to manipulate particles or cells, including optical tweezers (Diekmann et al., 2016; Zhang and Liu, 2008), magnetic tweezers (Carlsen et al., 2014; de Lanauze et al., 2013), acoustic tweezers (Hammarstrom et al., 2012; Gutierrez-Ramos et al., 2018), and HBO (Wu et al., 2018).

### 4.1 Light

A genetic circuit for dynamically planning bacterial lifestyles (planktonic, biofilm, or lytic) was created by a research team. By grading and modifying the illumination power density (LPD) of NIR light, the circuit precisely regulates the adhesion, colonisation, and drug release processes of bacteria during tumour treatment. The deep-tissue penetration of near-infrared technology provides a new strategy to spatiotemporally and noninvasively control bacterial genetic circuits *in vivo* (Fu et al., 2023). In addition, researchers have installed a “light-controlled biological switch” in bacteria, which can sense near-infrared light by embedding a photosensitive protein (a chimeric phytochrome-activated diguanylyl cyclase). When the near-infrared light irradiates the tumour, the switch activates the bacterial “pharmaceutical assembly line” to produce immune activators and matrix-degrading enzymes on demand. The set of “photonic command systems” can also adjust the light intensity and time and precise control of drug production for the precise treatment of tumours to provide an innovative solution (Qiao et al., 2025).

### 4.2 Magnetic field

However, the utility of optical tweezers for in vivo applications is severely limited because they can be used only in transparent media. Furthermore, direct exposure to high-intensity laser radiation may cause light damage and photothermally or photochemically inactivate bioactive drugs. Magnetic tweezers can penetrate nontransparent media, and an alternating magnetic field serves as an ideal signal for bacterial manipulation because of its virtually limitless tissue-penetrating capability and superior biosafety (Sitti and Wiersma, 2020; Huang et al., 2021; Ho et al., 2011). Ma X et al. engineered *E. coli* with Fe_3_O_4_@lipid nanocomposites to create magnetic tumour-homing bacteria. The paramagnetic Fe_3_O_4_ nanoparticles accumulate in female mouse orthotopic colon tumours and induce heat production and protein lysis, thereby releasing the bacteria’s anti-CD47 nanobody cargo, which has strong antitumour effects and enhances both innate and adaptive immune responses (Ma et al., 2023).

### 4.3 Acoustic waves

Unfortunately, unfocused magnetic fields make it difficult for magnetic tweezers to locate targets accurately *in vivo*. Biological samples that have been magnetically labelled may permanently lose their viability and bioactivity. Acoustic tweezers hold promise for handling biological particles because of their great tissue penetrability, minimal degree of damage, high spatial precision, and adaptability to a variety of media (Ozcelik et al., 2018; Meng et al., 2019). Yang Y et al. developed a phased array holographic acoustic tweezers technology that utilises electronic manipulation of sound waves to improve the aggregation efficiency of engineered bacteria in a tumour, successfully achieving targeted therapy of tumours in animal models. This study provides ideas for the targeted presentation of tumours and biomedical applications of cells (Yang et al., 2023). Moreover, the release of engineered bacterial drug proteins to be controlled by focused ultrasound can be applied noninvasively to specific areas, such as solid tumours, by generating high heat. Controlling the state change of the thermal drive switch produces long-lasting therapeutic protein output, achieving precise release of the payload (Abedi et al., 2022). Chen Y et al. created ultrasonic-responsive bacteria that can regulate the expression of foreign genes using ultrasound to promote the expression of the IFN-*γ* gene and improve antitumour effectiveness (Chen et al., 2022). An ultrasound-induced transgene expression system with increased controllability and sensitivity was developed (Gao et al., 2024), and an ultrasound-visible engineered bacterium was developed by Yaozhang Yang et al. for use in tumour chemoimmunotherapy (Yang et al., 2024).

### 4.4 Hyperbaric oxygen

Despite the many therapeutic benefits of ultrasonic therapy, its use is fraught with difficulties because tumour depth and spread limit its ability to influence gene expression effectively. HBO can increase the oxygen pressure in blood and tissues, which may guide the facultative anaerobe (EcN) to present improved targeting ability toward hypoxic tumours during HBO treatment. On the other hand, HBO can also deplete dense ECM to enhance intratumoral penetration and accumulation of EcN. Therefore, HBO therapy strategies can promote the accumulation of bacteria in the tumour and improve the depth of bacterial penetration and delivery efficiency. Nevertheless, the specific mechanism of HBO therapy needs to be further clarified. The feasibility and safety of its clinical application also need to be further verified (Xu et al., 2024).

Engineered bacteria have been combined with optical, magnetic, and acoustic tweezers and HBO to precisely control multiple key steps in the process of bacterial treatment of tumours and to precisely control the release of therapeutic drugs, which can improve treatment effectiveness and reduce costs for future cancer patients.

## 5 Conclusion and challenges

A multipurpose platform, bacteria can be employed as a single medication in conjunction with various antitumour therapies, including immunotherapy, phototherapy, chemotherapy, RT, and life technology, to increase the effectiveness of antitumour treatment. In addition, researchers can spatiotemporally control the expression of bacterial genes and drug release to avoid drug release and adverse reactions in healthy tissues. Although bacterial cancer treatments have advanced significantly, safety and biocontainment issues must still be considered during the clinical translation process. Now, a randomised, double-blind clinical trial to evaluate the safety and human tolerance of the oral probiotic *streptococcus sali*var*ius* eK12 is in progress (NCT06380270). In patients with incurable hepatic spread from any non-hematologic source, a phase 1 study of an attenuated, IL-2-expressing *Salmonella typhimurium* has been finished (NCT01099631). Recently, a multiple myeloma trial of an orally administered *salmonella*-based survivin vaccine has also been completed (NCT03762291). In patients with metastatic pancreatic cancer receiving standard chemotherapy, a phase II clinical trial evaluates the effectiveness of various doses of oral administration of an attenuated strain of *Salmonella Typhimurium* expressing IL-2 (NCT04589234). Nevertheless, it has been demonstrated that host toxicity from live bacteria limits tolerated dose and efficacy, sometimes resulting in the termination of clinical trials (Toso et al., 2002; Leventhal et al., 2020; Flickinger et al., 2018; Sanders et al., 2010; Yelin et al., 2019).

As bacterial systems used for cancer treatment continue to enter clinical trials, the outcomes of these trials will dictate future engineering methods to improve their efficacy. In addition, engineered bacterial therapy still faces many challenges, especially safety issues. All bacterial components, live bacteria, and bacterial communities are antigenic substances to the human body, and their presence is bound to cause an immune response. Strong immune reactions, such as cytokine storms and bacteraemia, can be induced by excessive immunological responses, which can potentially endanger a patient’s life. An engineered bacterial therapy platform with dual capabilities—customizable therapeutic outputs and exact dosage regulation—would serve to overcome this current restriction. For example, Longliang Qiao’ team developed an engineered bacterium based on *Salmonella enteritidis*, which demonstrated promising anti-tumour effects and therapeutic potential through a light-triggered antibody *in situ* synthesis mechanism, while significantly reducing the risk of toxic cytokine storm in peripheral blood (Qiao et al., 2025). The genetic instability of bacteria is also a potential issue since mutations might result in harmful or inefficient phenotypes. Furthermore, achieving precise *in vitro* control is also a major challenge because the degree of artificial change can be limited by the bacteria’s structure and function, which prevents the full customisation of synthetic materials. Furthermore, the selection of exogenous stimulus responses is limited since the working environment of engineered bacteria must be similar to the living habitat of the bacterium. In addition to these, the effectiveness of engineered bacteria is in dire need of improvement. Lin S et al. enhance phage therapy by coating single bacteriophage-infected bacteria with polymer to preserve phage vitality (Lin et al., 2025). The development of synthetic biology is expected to solve these problems. With the deepening of research regarding bacteria-mediated cancer treatment strategies, we speculate that in the future, more antitumour methods will be combined with engineered bacteria or that a variety of antitumour therapies will be integrated, which will result in the development of more cancer treatment methods with efficient, safe and personalized characteristics. Cross-cutting research can also provide unintended benefits, such as an engineered bacterial bioreactor for tumour therapy via a Fenton-like reaction with localized hydrogen dioxide generation (Fan et al., 2019). These work will establish a robust theoretical framework and serve as a valuable reference to facilitate advancements in research and clinical application within this field.

## Glossary

BNPs: Bi_2_S_3_ nanoparticles
Bac@BNP: Engineered bacteria (Bac) and Bi_2_S_3_ nanoparticles (BNPs)
TME: Tumour microenvironment
CAR: Chimeric antigen receptor
*E. coli* Nissle 1917: *Escherichia coli* Nissle 1917
ROS: Reactive oxygen species
ASD: aspartate semialdehyde dehydrogenase
YB1: a rationally designed “obligate” anaerobic *Salmonella typhimurium* strain
RT: Radiotherapy
OMVs: Outer-membrane vesicles
IRT: Internal radioisotope therapy
^125^I-VNP/^131^I-VNP: Inactivated bacteria vector with ^125^I/^131^I labelling
*S. typhimurium*: *Salmonella Typhimurium*
DOX: Doxorubicin
NanoBEADS: Nanoscale bacteria-enabled autonomous drug delivery system
PDA: Polydopamine
OMVs: Outer membrane vesicles
EcN-ca-Dox: Doxorubicin (DOX) is conjugated onto EcN in the current study via acid-labile linkers of cis-aconitic anhydride
NK: Natural killer
TLR-4: Toll-like receptor 4
TLR-5: Toll-like receptor 5
FlaB: Flagellin B
STING: STimulator of Interferon Genes
APCs: Antigen-presenting cells
*E. coli*: *Escherichia coli*
EcN: *Escherichia coli* Nissle 1917
IFN-I: Type I Interferon
PDMN: Polydopamine
*S. epidermidis*: *Staphylococcus epidermidis*
NKT cell: Natural killer T-cell
IFN-*γ*: Interferon-γ
CD47nb: Nanobody antagonist of CD47
TILs: Tumour-infiltrating lymphocytes
BNPs: Bacterial membrane-coated nanoparticles
PTT: Photothermal therapy
PDT: Photodynamic therapy
PTA: Photothermal agent
PD-1: Programmed cell death 1
NIR: Near-infrared
PA: Photoacoustic
TNF-*α*: Tumour necrosis factor-α
PPSB: Purple photosynthetic bacteria
NPNs: Nanopathogens
PSs: Photosensitisers
*C. butyricum*.: *Clostridium butyricum*
d-Ala: D-alanine
TPApy: Photosensitiser
d-Ala-TPAPy: D-alanine-photosensitiser
PS: Photosensitiser
TRAIL: Tumour necrosis factor-related apoptosis-inducing ligand
BP: Black phosphorus
NPs: Nanoparticles
Ir-HEcN: Iridium (III) photosensitiser-bacteria hybrid
HBO: Hyperbaric oxygen
LPD: Lighting power density
OVs: Oncolytic viruses
OAs: Oncolytic adenoviruses
Ep-AH: Azurin and hlpA genes were developed using *Escherichia coli* Nissle 1917 (EcN) chassis
CRC: Colorectal cancer
